# Primary Medication Non-Adherence after Discharge from a General Internal Medicine Service

**DOI:** 10.1371/journal.pone.0061735

**Published:** 2013-05-02

**Authors:** Brooks A. Fallis, Irfan A. Dhalla, Jason Klemensberg, Chaim M. Bell

**Affiliations:** 1 Department of Medicine, University of Toronto, Toronto, Ontario, Canada; 2 Institute of Health Policy, Management and Evaluation, University of Toronto, Toronto, Ontario, Canada; 3 Department of Medicine, St. Michael's Hospital, Toronto, Ontario, Canada; 4 Li Ka Shing Knowledge Institute, St. Michael's Hospital, Toronto, Ontario, Canada; 5 Institute for Clinical Evaluative Sciences, Toronto, Ontario, Canada; 6 Department of Medicine, Mount Sinai Hospital, Toronto, Ontario, Canada; Universidad Peruana Cayetano Heredia, Peru

## Abstract

**Background:**

Medication non-adherence frequently leads to suboptimal patient outcomes. Primary non-adherence, which occurs when a patient does not fill an initial prescription, is particularly important at the time of hospital discharge because new medications are often being prescribed to treat an illness rather than for prevention.

**Methods:**

We studied older adults consecutively discharged from a general internal medicine service at a large urban teaching hospital to determine the prevalence of primary non-adherence and identify characteristics associated with primary non-adherence. We reviewed electronic prescriptions, electronic discharge summaries and pharmacy dispensing data from April to August 2010 for drugs listed on the public formulary. Primary non-adherence was defined as failure to fill one or more new prescriptions after hospital discharge. In addition to descriptive analyses, we developed a logistical regression model to identify patient characteristics associated with primary non-adherence.

**Results:**

There were 493 patients eligible for inclusion in our study, 232 of whom were prescribed new medications. In total, 66 (28%) exhibited primary non-adherence at 7 days after discharge and 55 (24%) at 30 days after discharge. Examples of medications to which patients were non-adherent included antibiotics, drugs for the management of coronary artery disease (e.g. beta-blockers, statins), heart failure (e.g. beta-blockers, angiotensin converting enzyme inhibitors, furosemide), stroke (e.g. statins, clopidogrel), diabetes (e.g. insulin), and chronic obstructive pulmonary disease (e.g. long-acting bronchodilators, prednisone). Discharge to a nursing home was associated with an increased risk of primary non-adherence (OR 2.25, 95% CI 1.01–4.95).

**Conclusions:**

Primary non-adherence after medications are newly prescribed during a hospitalization is common, and was more likely to occur in patients discharged to a nursing home.

## Introduction

Medication adherence is the degree to which a patient takes a medication as prescribed. Non-adherence to prescription medications is associated with a higher risk of death, more frequent hospitalizations, and greater health care expenditure.[1]–[5] The reasons for medication non-adherence are varied and include treatment, patient, and health system factors.[6]–[8]

Most research in this area has focused on secondary non-adherence, which occurs when a patient discontinues a medication after filling the initial prescription. There is far less data about primary non-adherence, which occurs when a patient does not fill an initial prescription. Recent studies in the outpatient primary care setting using large administrative databases found primary non-adherence rates ranging from 7% to 24%.[9]–[11] When looking specifically at the management of certain chronic medical conditions such as diabetes, hypertension and dyslipidemia, nearly one out of every three patients was primarily non-adherent.[10], [11]

In contrast, primary non-adherence at the time of hospital discharge has not been well studied. This is a particularly important time for medication adherence since new medications may be initiated during a hospitalization, and may be continued at discharge. We therefore sought to determine the prevalence of primary non-adherence and determine associated characteristics.

## Methods

### Overview

We identified consecutively discharged adults aged 66 or older from the general internal medicine service at St. Michael's Hospital to determine if new prescriptions were provided to the patient and whether the prescription was dispensed soon after hospital discharge. The St. Michael's Hospital Research Ethics Board approved the study protocol (REB#10–300) and waived the need for individual consent for any patients. This was a retrospective study so individual consent would have been difficult. The information was thought to be important and the study low risk.

### Setting

St. Michael's Hospital is a large, inner city, academic hospital in Toronto, Canada. The 70 bed general internal medicine service discharged 3499 patients in 2010. Five clinical teaching teams are composed of an attending hospitalist physician, a group of housestaff (a second- or third-year internal medicine resident, zero to 2 first-year residents, and medical students in their penultimate or final year of medical school), a pharmacist, and allied health staff. Housestaff are responsible for producing electronic discharge summaries and prescriptions for their patients.

### Data sources

#### New prescriptions and baseline data

Patient discharge summaries were accessed from the St. Michael's Hospital online medical record discharge system, eDischarge.[12] Data collected from eDischarge included demographic data (date of birth, age, gender), admission data (admission/discharge date, admission diagnosis, length of stay, discharge destination) and medication data (total number of medications, new medications, dose, duration, standing vs. PRN, and route of administration). eDischarge has a medication reconciliation section which prompts housestaff to label each medication as “new” or “changed” or “unchanged”. The default setting is blank and therefore “new” must be actively selected by the housestaff completing the electronic prescription. If the medication reconciliation section was left blank it was not clear which medications were new and therefore these patients were not included in our analysis. This section is incorporated into the automatically generated electronic prescription that is provided to the patient upon discharge.

#### Adherence data

Medication dispensing data were obtained using a web-accessible interface that allows qualified health care professionals to view publicly funded prescription drug claims for Ontario residents that were dispensed within 1 year of the viewing date. All Ontarians aged 65 and older have their medications funded publicly. Outpatients and nursing home residents have their drugs covered by the same payer and are displayed in the same manner in the Drug Profile Viewer. Some individuals also have private insurance for prescription drugs, but private insurance generally functions as a payer of last resort. At discharge, our institution does not pay for medications for patients who are 65 or older because these patients are covered by the Ontario Drug Benefits plan and there is an outpatient pharmacy on site. The Drug Profile Viewer provides for each drug the name, dose, duration, frequency, and date dispensed. The database that served as the source of medication adherence data for the study has been used in previous studies and has been demonstrated to have excellent accuracy [3], [13], [14].

#### Post-discharge healthcare utilization data

Patient visit data in the St. Michael's Hospital patient tracking system were viewed to ascertain whether a patient had been seen in the emergency department or readmitted to the hospital within 30 days of discharge.

### Study population

**Figure 1:**
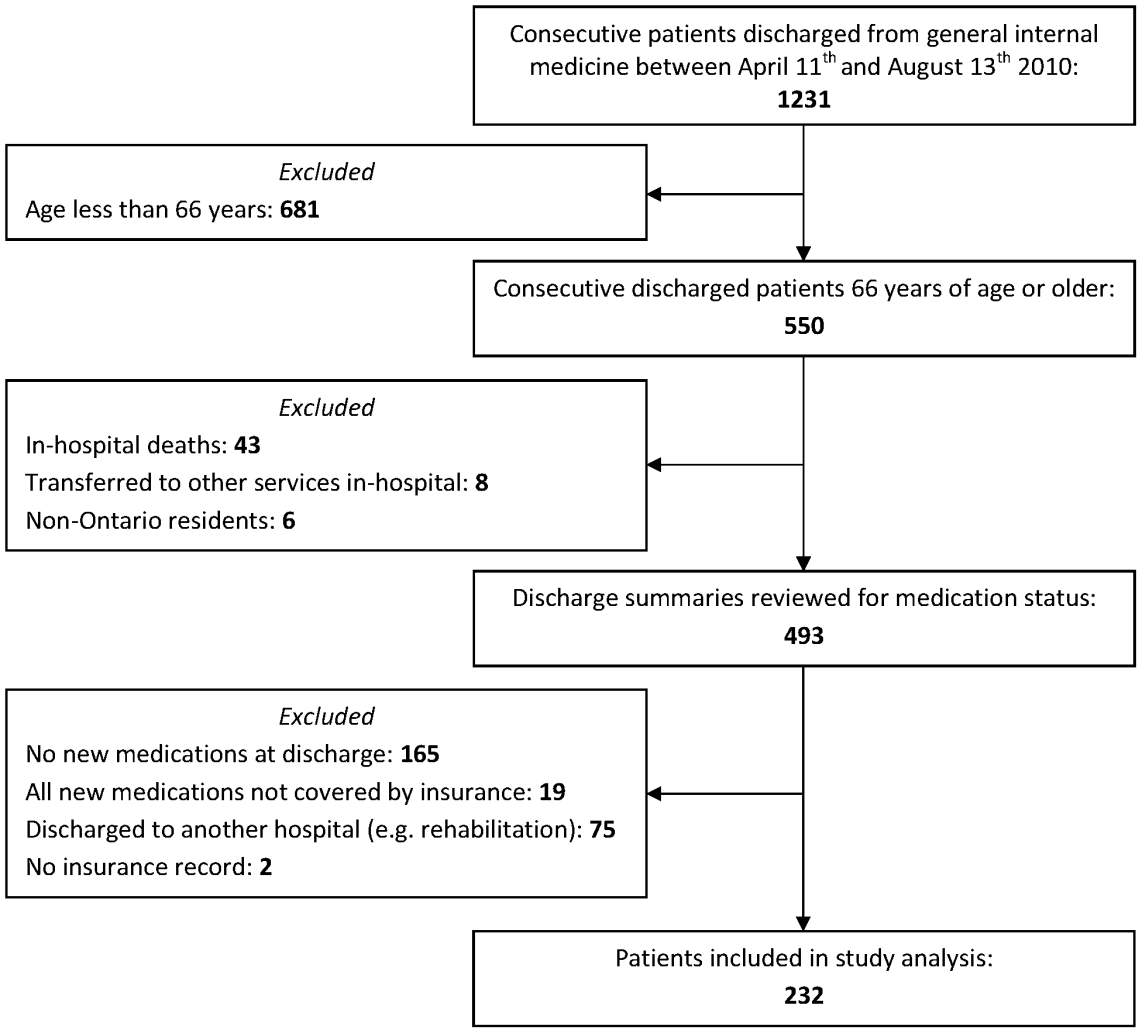
Identification of study patients.

We included consecutive patients discharged from the general internal medicine service at St. Michael's Hospital between April 2010 and August 2010 (Figure 1). We chose to include patients 66 and older so that dispensing data from the previous year could be used to confirm that new medications were indeed novel for that patient. We excluded patients who were transferred to another hospital (including rehabilitation facilities), patients who were not prescribed any new medications at the time of discharge, and patients who were only newly prescribed medications that are not reimbursed by the public payer in Ontario. Examples of these include aspirin, gabapentin and vitamin D. We also excluded patients who had no record of ever having received a publicly funded medication because the inclusion of these patients would have lead to erroneous overestimation of the rate of primary medication non-adherence.

### Outcomes

The primary outcome was primary non-adherence at 7 days. Non-adherence was defined as failing to fill one or more new prescriptions within 7 days of hospital discharge. Patients readmitted to hospital or returning to the emergency department within 7 days of discharge and prior to medication dispensation were deemed non-adherent. As a secondary outcome, we also analysed data at 30 days after discharge.

We separately analyzed primary non-adherence for medications we deemed to be of higher and lower importance. “High importance” medications included antibiotics, drugs for the management of coronary artery disease, heart failure, stroke, diabetes, chronic obstructive pulmonary disease, osteoporosis, and primary or secondary prevention of gastrointestinal bleeding. Medications deemed to be of lower importance included medications used to treat symptoms such as gastroesophageal reflux, constipation, nausea and pain, supplements such as iron and folate and topical rash therapies. Information on new medication indication was ascertained from the electronic discharge summary. A free-text field indicating medication indication on the e-prescription was the primary source. If this field was left blank, the main text of the discharge summary and admission diagnosis was reviewed to decide indications for the newly prescribed drug. In a small number of cases, the indication remained unclear after primary review. In these cases, the three physician co-authors discussed the case and reached a consensus.

### Analysis

In addition to descriptive analyses, we conducted univariate analyses to determine which factors were associated with primary non-adherence. Using the results of the univariate analysis we selected factors which may be associated with primary non-adherence. We used these covariates to develop a logistic regression model to determine which factors, if any, were independently associated with primary non-adherence in our study population. SAS® 9.3 software was used for statistical analysis.

## Results

There were 493 seniors who were eligible for inclusion and 232 patients were prescribed new medications.(Figure 1) The most common admitting diagnoses for the 232 analysed patients were infections (e.g. pneumonia, urinary tract infection, cellulitis, other), acute exacerbations of chronic obstructive pulmonary disease, acute decompensated heart failure, stroke or transient ischemic attack, gastrointestinal hemorrhage, and various malignancy-related conditions. These accounted for approximately two-thirds of admission diagnoses.

There were 197 patients discharged home and 35 discharged to a nursing home (Table 1). The average age was 78 and slightly more than half were female. The median hospital length of stay was 6 days (interquartile range 4 to 9). The median number of discharge medications was 10 (interquartile range 7 to 13.25). On average, 2.1 medications were newly prescribed at discharge.

**Table 1:** Descriptive characteristics of study patients.

Total number of patients (n,%)	232
**Age** (mean±Sd*)	78±7.9
**Sex** (n,%)	
Male	113 (49%)
Female	119 (51%)
**All discharge medications** (median, IQR^+^)	
All	10 (7–13.25)
Standing	9 (6–12)
PRN	1 (0–2)
**New discharge medications** (mean±Sd)	
All	2.1±1.5
Standing	1.8±1.3
PRN	0.3±0.6
**Standing vs. PRN for new medications** (n,%)	
Standing & PRN	44 (19%)
Standing only	177 (76%)
PRN only	11 (5%)
**Length of stay** (median, IQR)	6 days (4–9)
**Discharge destination** (n,%)	
Home	197 (85%)
Long-term care	35 (15%)
PCP^**^ copied on discharge summary (n,%)	195 (84%)
**Patients readmitted** (n,%)	
ED^++^ or Hospital within 30 days	63 (27%)
Hospital within 30 days	42 (18%)

* Sd  =  standard deviation. ^+^ IQR  =  interquartile range. ^**^ PCP  =  Primary care physician. ^++^ ED  =  Emergency department.

Overall, we found that 66 patients (28%) at 7 days and 55 patients (24%) at 30 days exhibited primary non-adherence (Table 2). There were no significant demographic differences between the adherent and non-adherent groups in terms of age, gender, number of medications and length of stay. Including the name of the primary care physician on the discharge summary (which would result in the hospital sending the discharge summary to this physician) was not associated with a higher rate of adherence (Table 3). When we focused only on “high importance” medications, the patient non-adherence rate was 20% at 7 days after hospital discharge and 16% at 30 days. Further, at 30 days after discharge 62 (27%) patients had an unscheduled return to hospital (ED or readmission) and 42 (18%) were readmitted. At 7 days after discharge 20 patients were re-admitted and 3 of these patients were categorised as non-adherent.

**Table 2:** Characteristics of study patients divided by primary medication adherence at 7 days after discharge.

	Adherent at 7 days	Non-adherent at 7 days
Total number of patients (n,%)	166 (72%)	66 (28%)
**Age** (mean±Sd*)	77.7±8.0	78.8±7.6
**Sex** (n,%)		
Male	79 (48%)	34 (52%)
Female	87 (52%)	32 (48%)
**All discharge medications** (median, IQR^+^)		
All	10 (7–13.75)	10.5 (8.25–13)
Standing	9 (6–12.75)	10 (7.25–12)
PRN	1 (0–1)	1 (0–2)
**New discharge medications** (mean±Sd)		
All	2.0±1.5	2.4±1.5
Standing	1.7±1.3	2.0±1.3
PRN	0.2±0.5	0.5±0.7
**Standing vs. PRN for new medications** (n,%)		
Standing & PRN	25 (15%)	19 (29%)
Standing only	134 (81%)	43 (65%)
PRN only	7 (4%)	4 (6%)
**Length of stay** (median, IQR)	5 days (4–8)	7 days (4–11)
**Discharge destination** (n,%)		
Home	146 (88%)	51 (77%)
Long-term care	2 (12%)	15 (23%)
PCP^**^ copied on discharge summary (n,%)	138 (83%)	57 (86%)
**Patients readmitted** (n,%)		
ED^++^ or Hospital within 30 days	49 (30%)	14 (21%)
Hospital within 30 days	34 (20%)	8 (12%)

* Sd  =  standard deviation. ^+^ IQR  =  interquartile range. ^**^ PCP  =  Primary care physician. ^++^ ED  =  Emergency department.

**Table 3:** Prescription adherence at 7 and 30 days after hospital discharge.

	Filled prescription at 7 d (n,%)	Filled prescription at 30 d (n,%)
**All patients** (n = 232)		
Adherent*	166 (72%)	177 (76%)
Non-adherent*	66 (28%)	55 (24%)
**Patients prescribed 1 new standing medication** (n = 110)		
Adherent	83 (75%)	89 (81%)
Non-adherent	27 (25%)	21 (19%)
**Patients prescribed 2 new standing medication** (n = 62)		
Adherent	47 (76%)	48 (77%)
Non-adherent	15 (24%)	14 (23%)
**Patients prescribed 3 or more new standing medications** (n = 49)		
Adherent	29 (59%)	33 (67%)
Non-adherent	20 (41%)	16 (33%)
**Patients prescribed one or more "important" medications ^+^**		
Adherent ^**^	186 (80%)	195 (84%)
Non-adherent	46 (20%)	37 (16%)

* Adherent patients picked up all medications, while non-adherent patients failed to pick-up at least 1 medication. ^+^“Important” medications exclude PRN, symptom control, dermatological, and supplements. ^**^Adherent to “important medications means either fully adherent or non-adherent to medications not classified as "important".

A total of 488 new prescriptions were provided to the 232 patients. A total of 100 newly prescribed drugs went unfilled by a total of 66 different patients at 7 days after discharge (Table 4). The total prescription non-adherence rate was therefore 21% (100 out of 488). “High importance” examples of primary non-adherence included antibiotics (e.g. for pneumonia, acute exacerbations of COPD, urinary tract infections, *Clostridium difficile* colitis and cellulitis), drugs for the management of coronary artery disease (e.g. beta-blockers and statins), heart failure (e.g. beta-blockers, angiotensin converting enzyme (ACE) inhibitors and furosemide), stroke (e.g. statins and clopidogrel), diabetes (e.g. insulin), chronic obstructive pulmonary disease (e.g. long-acting bronchodilators and prednisone), and proton pump inhibitors for primary or secondary prevention of gastrointestinal bleeding. A total of 60 prescriptions for “high importance” medications went unfilled by 46 different patients. The “high importance” prescription non-adherence rate at 7 days was therefore 18% (60 out of 339).

**Table 4:** Medications to which patients exhibited primary non-adherence.

Drug Category and indication	Prescriptions	Specific Medications (n)
**Antibiotics**		
Pneumonia	4	Levofloxacin (2), azithromycin (1), cefuroxime (1)
COPD exacerbation	2	Azithromycin (1) cefuroxime (1)
Cellulitis	5	Cephalexin (1), ciprofloxacin (1), cloxacillin (2), levofloxacin (1)
Urinary tract infection	5	Ciprofloxacin (5)
*Clostridium difficile* colitis	2	Metronidazole (1), vancomycin (1)
Other *	5	Amoxicillin (1), clarithromycin (1), dapsone (1), ofloxacin eye drops (1), valacyclovir (1)
**Proton-pump inhibitors**		
Upper gastrointestinal bleed	4	Pantoprazole (1), omeprazole (2), pantoprazole (1)
Gastrointestinal bleed prophylaxis	2	Pantoprazole (2)
Gastroesophageal reflux	2	Pantoprazole (1), rabeprazole (1)
**Cardiac & Vascular**		
Stroke (secondary prevention)	5	Atorvastatin (3), clopidogrel (2)
Coronary artery disease (secondary prevention)	4	Atorvastatin (2), metoprolol (1), nitroglycerin SL (1)
Heart failure	3	Bisoprolol (1), ramipril (1), furosemide (1)
Venous thromboembolism	1	Warfarin (1)
**Respiratory**		
Chronic obstructive pulmonary disease	8	Prednisone (3), salbutamol (3), Advair discus (1), tiotropium (1)
**Endocrine**		
Diabetes mellitus	2	Humalog (1), Humulin 30/70 (1)
Osteoporosis	1	Risedronate (1)
Other	2	Fludrocortisone (1), leuprolide acetate (1)
**Symptom management**		
Nausea	4	Ondansetron (4)
Constipation	14	Sennosides (6), docusate sodium (4), lactulose (4)
Pain	8	Hydromorphone (3), morphine (3), oxycodone & acetaminophen (2)
Dementia associated behavioural problems	4	Risperidone (2), quetiapine (1), trazodone (1)
**Other**		
Nutritional supplements (iron, folate)	5	Ferrous fumarate (2), ferrous gluconate (1), folate (2)
Topical dermatologic treatments	3	Betamethasone valerate (1), hydrocortisone acetate and urea (1), mupirocin (1)
Other ^+^	5	Acetazolamide (1), colchicine (1), primidone (1), tamsulosin (1), verapamil (1)
**TOTAL**	**100**	

* Other antibiotics: PCP prophylaxis (dapsone), H.pylori (amoxicillin & clarithromycin), herpes zoster (valacyclovir), eye infection (ofloxacin drops). ^+^ Other: Migraines (verapamil), tremor (primidone), benign prostatic hypertrophy (tamsulosin), bicarbonate (acetazolamide), gout (colchicine).

Patients who were discharged to long-term care had higher rates of primary non-adherence (43%) compared to those patients discharged to a home environment (26%). This difference was statistically significant in our multivariate regression model (OR 2.25, 95% CI 1.01–4.95). Other patient characteristics were not associated with non-adherence.

## Discussion

We examined the rate of primary non-adherence in patients discharged from a general internal medicine service. We found that 28% of patients exhibited primary non-adherence one week after discharge and one-fifth of all new prescriptions went unfilled. Restricting our analysis to important drugs such as antibiotics, drugs for cardiovascular risk reduction, and gastrointestinal hemorrhage management showed similar results. Patients discharged to a nursing home were more likely to be non-adherent than patients discharged to their own homes.

The results of our study are in agreement with previous outpatient studies which suggest that primary non-adherence is a significant issue. A primary care audit found that 14.5% of patients did not fill a prescription and that 5.2% of prescriptions went unfilled.[15] Recent studies in the outpatient primary care setting using large administrative databases found primary non-adherence rates ranging from 7% to 24%.[9]–[11] One previous study examining non-adherence after hospital admission found a 27% non-adherence rate after acute myocardial infarction.[4] The authors of this study identified a significantly increased risk of mortality associated with non-adherence. This is one of the few studies to assess adherence with prescriptions on discharge from hospital but it did not distinguish between all discharge medications and those which were new for each patient.

A strength of our study is that we report on a broad population from a general internal medicine service. Also, by examining individual discharge summaries as well as previous medication dispensing records we were able to specifically identify prescriptions which were for new medications and therefore we were able to isolate primary non-adherence. Primary medication non-adherence occurred despite the significant life event of a hospital admission and the interdisciplinary nature of the general internal medicine service at a teaching hospital. Our study demonstrates that primary non-adherence is not limited to the primary care setting and emphasises that hospital discharge is an important time to be aware of the potential for primary medication non-adherence.

There were many different classes of drugs for which patients were non-adherent. Many patients did not fill medications for constipation or analgesia, which although less significant from a disease management perspective, can carry significant morbidity. Particularly striking examples of non-adherence with drugs for management of acute conditions included pantoprazole following a gastrointestinal hemorrhage, prednisone and antibiotics for the management of acute exacerbations of chronic obstructive pulmonary disease (COPD), vancomycin and metronidazole for *Clostridium difficile* colitis, and furosemide for the management of heart failure. In addition, there were many examples of primary non-adherence with drugs prescribed for the management of chronic medical conditions. These included inhaled steroids and anticholinergics for COPD, statins after stroke or in the context of coronary artery disease, and ramipril and bisoprolol in the chronic management of heart failure.

We were surprised that patients discharged to nursing homes were more likely to be non-adherent than patients discharged home. In some instances, nursing homes may not have robust systems in place to ensure that the discharge prescription is rapidly approved by the nursing home physician and then sent to a pharmacy. Previous studies suggest there is need for improved communication between hospitals and nursing homes at the time of discharge to aid implementation of discharge medications.[16]

Our study has several limitations that merit emphasis. First, our study was relatively small and was conducted at only one site. Therefore, the generalizability of our findings is uncertain. Second, we used data from discharge summaries and administrative data rather than speaking with residents or patients directly or reviewing chart notes. However, the administrative database we used has been well validated and the discharge summaries were in almost all instances unambiguous about the reason a particular medication was prescribed. Third, we were unable to assess adherence to medications that are not publicly funded in Ontario. Still, the formulary is broad and includes most important medications.

Regarding the significantly greater rates of primary non-adherence in patients discharged to nursing homes, there may be intentional and reasonable decisions made by clinicians at the long-term care facilities to not follow the hospital discharge instructions and prescriptions because of greater familiarity with patient directives and goals of care. However, it is also plausible that the processes of care in some nursing homes are not sufficiently robust to minimize the rate of non-adherence. Given the borderline statistical significance of this association in our study, and the fact that this was not a pre-specified hypothesis, further studies could focus on confirming this lower adherence rate and investigating factors which may be implicated.

Lower health literacy, lower cognitive function and an increased number of prescribed medications have been associated with lower patient understanding of their medication regimen.[17] Future work in the area of medication primary non-adherence should examine aspects of communication and health literacy and how they impact primary non-adherence.

Our study identifies discharge from hospital as a high-risk period for non-adherence. This could relate to poor communication at the time of discharge between the inpatient medical team, patient, primary care physician, and nursing homes. Patients may not be aware they are supposed to fill a prescription for new medications, or the importance of the new medications is not adequately emphasized at the time of discharge and therefore patients elect not to fill the medications. Since hospital discharge is a time where new medications are commonly introduced, it should also be a time to communicate medication regimens and reinforce adherent medication taking behaviour. Additional strategies to minimize non-adherence after hospital discharge should also be developed and evaluated.
